# Preparation and Characterization of a New Bis-Uracil Chitosan-Based Hydrogel as Efficient Adsorbent for Removal of Anionic Congo Red Dye

**DOI:** 10.3390/polym15061529

**Published:** 2023-03-20

**Authors:** Rana A. Alharbi, Fahad M. Alminderej, Nouf F. Al-Harby, Noura Y. Elmehbad, Nadia A. Mohamed

**Affiliations:** 1 Department of Chemistry, College of Science, Qassim University, Buraidah 51452, Saudi Arabia; 2 Department of Chemistry, Faculty of Science and Arts, Najran University, Najran 55461, Saudi Arabia; 3 Department of Chemistry, Faculty of Science, Cairo University, Giza 12613, Egypt

**Keywords:** chitosan, crosslinking, CR dye, adsorption, hydrogels, adsorption kinetic, adsorption thermodynamic, adsorption isotherm

## Abstract

A new hydrogel, based on chitosan crosslinked with 2-chlorophenyl-bis(6-amino-1,3-dimethyluracil-5-yl) methane, (2Clph-BU-Cs), has been successfully created. Various instrumental techniques such as elemental analysis, FTIR, SEM, and XRD were used to prove its structure. Its removal efficiency for anionic Congo red (CR) dye under different conditions for industrial wastewater treatment was studied. For optimizing the conditions to maximize CR dye removal, the impacts of temperature, contact time, pH, and initial concentration of the dye on adsorption capacity were investigated. The removal of the dye was pH-dependent, with a much higher value achieved at pH 4 than at pH 7 and 9. The maximum adsorption capacity of the hydrogel was 93.46 mg g^−1^. The model of adsorption process was fitted to the pseudo-second-order kinetic model. The intraparticle diffusion demonstrated the multi-step nature of the adsorption process. The thermodynamic results showed that the adsorption process was endothermic because of the positive value of enthalpy (43.70 kJ mol^−1^). The process of adsorption at high temperatures was spontaneous, according to the values of ∆G^0^. An increase in randomness was seen in the value of ∆S°. Generally, the investigated hydrogel has the potential to be used as a promising effective reusable adsorbent for industrial wastewater remediation.

## 1. Introduction

Since water is a resource on which almost all life on earth depends, its demand has sharply increased as a result of population growth and industrial development, which have a negative impact on its quality. The wastewater produced by the textile sector in particular is one of the most dangerous factors harming the environment and public health. This results from the widespread use of synthetic dyes, which are not only thermally and chemically stable but also highly poisonous, mutagenic, carcinogenic, and poorly biodegradable [1].

**Figure 1:**
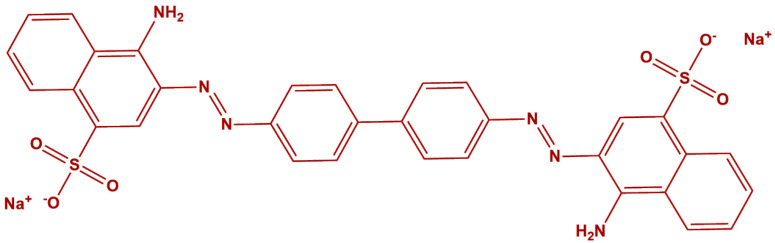
Chemical Structure of the CR dye.

Congo red (CR) dye (Figure 1), which contains azo groups with aromatic rings and sulphonate groups, is a highly toxic dye that is utilized in a variety of industries. As a result, it is critical to eliminate this life-threatening chemical from wastewater prior to their final disposal into the environment [2,3,4].

To reduce or eliminate harmful contaminants from aquatic solutions, several strategies have been used including solvent extraction, magnetic and membrane separation, ion exchange, precipitation, oxidation, photochemical reactions, irradiation, bacterial treatment, and reduction [5,6,7,8]. Recently, the adsorption technique for the pollutant’s removal has attracted much attention since it is characterized by high effectiveness, easy implementation, simplicity of operation, and low cost in addition to ecological safety [9,10,11].

Chitosan (Cs) is one of the most prevalent natural amino-polysaccharides and one of the most promising organic materials. It has drawn much attention due to its distinctive properties and biological activities. It has exceptional features like renewability, bio-degradability, bio-compatibility, non-toxicity, antimicrobial activity, and anionic dyes adsorption [12,13,14,15,16,17,18]. The disadvantages of Cs include its high solubility in acidic media, as well as its low heat resistance and restricted porosity, resulting in a significant obstacle for many of its uses. These drawbacks could be addressed utilizing several chemical modification processes of Cs such as grafting using a functional monomer [19], blending with a synthetic or natural polymer [20,21], crosslinking by creation of bridges between its chains [22,23] and substitution reaction using a functional compound [24,25].

Uracil is a naturally occurring component and a member of the pyrimidine family [26], and one of the four nucleobases of the biopolymer RNA [26,27,28,29]. The uracil unit is a portion of the building nine blocks of RNA, DNA, and other natural products, thus, it is one of the most significant structures in life [30]. Uracil creates two hydrogen bonds with adenine in RNA. In DNA, thymine replaces uracil as the nucleobase. It can be thought of as a demethylated version of thymine [26]. It has demonstrated a class of molecules that continues to attract medicinal chemists, organic chemists, and photobiologists [31]. In our previous work, a uracil-modified chitosan-based adsorbent has shown high adsorption efficiency for removal of CR dye from its aqueous solution [4].

Accordingly, in the current study, 2-chlorophenyl-bis(6-amino-1,3-dimethyluracil-5-yl) methane (2Clph-BU), that was synthesized by condensing 6-amino-1,3-dimethyl uracil with 2-chloro benzaldehyde, has been used for crosslinking the chitosan chains to decrease its solubility in acidic media since the effluents are often acidic (pH range from 6.5 to 8.5), to improve its capacity for adsorption of anionic CR dye from aqueous medium for the treatment of industrial wastewater. This was accomplished using a four-step reaction; in the first step, chitosan amino groups were protected by turning them into imine groups by reacting chitosan with benzaldehyde, then, in the second step, epoxy moieties were incorporated into the -OH groups on the C6 of the formed chitosan Schiff base (CsSB) via a reaction with epichlorohydrin. In the third step, the epoxide moieties were simply opened using the prepared 2Clph-BU via a free pair of electrons of its -NH_2_ groups to obtain 2Clph-BU crosslinked chitosan Schiff bases (2Clph-BU-CsSB), and finally, the protection of the chitosan’s amino groups was eliminated using acidic medium to obtain 2Clph-BU-Cs. Incorporating nitrogen-rich 2Clph-BU connections between the chitosan repeating units, in addition to the regained amnio groups, would result in an increase in the number of basic centers accessible for adsorbing the anionic CR dye. Further, the electron withdrawing nature of the chloro-substituent group would decrease the electron density on 2Clph-BU-Cs adsorbent, and consequently enhance its performance for anionic CR dye adsorption. To identify the ideal adsorption conditions and increase its capacity, the effects of several variables on the adsorption process, such as temperature, initial concentration of dye, contact time, and adsorption medium pH, have been studied. In addition, some adsorption process’s thermodynamic parameters such as enthalpy, free energy, and entropy were also investigated.

## 2. Materials and Methods

### 2.1. Materials

Chitosan (1.0–3.0 × 10^5^ g mol^−1^ molecular weight, 98% degree of deacetylation, purity 98%, and ash ≤ 2%) was made from the shells of shrimps and crabs Pandalus borealis and purchased from Acros Organics (Morris Plains, NJ, USA). Methanol (HPLC grade) was supplied by chem-lab, (Zedelgem, Belgium). Ethanol, epichlorohydrin, and hydrochloric acid were obtained from PanReac. AppliChem- ITW Reagent (Darmstadt, Germany ). Sodium carbonate and sodium hydroxide pellets were obtained from Loba Chemie, (Mumbai, India). Six-amino-1,3-dimethyluracil, benzaldehyde, 2-chlorobenzaldehyde, were purchased from Sigma-Aldrich, (Munich, Germany). Congo red dye (Figure 1) was supplied by Winlab (Leicestershire, UK). The buffer powder pillows of pH 4, 7 and 9 were supplied by Hach Company (Loveland, CO, USA). 

### 2.2. Methods

#### 2.2.1. Synthesis of 2Clph-BU

**Scheme 1:**
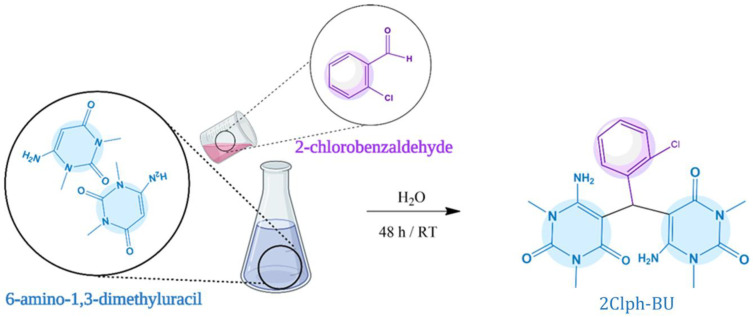
Preparation of 2Clph-BU.

Six-amino-1,3-dimethyluracil (155 mg, 1 mmol) was dissolved in 30 mL of distilled water. To such a solution, 2-chlorobenzaldehyde (70 mg, 0.5 mmol) was gradually added and the resultant mixture stirred at room temperature (RT). The product (2Clph-BU) started to appear as a white precipitate after a short period, and stirring was continued for 48 h to ensure that all of the reactants were transformed into a product (yield = 99.00%). The white precipitate was filtered, washed repeatedly with water and dried (Scheme 1) [29].

#### 2.2.2. Synthesis of a New 2Clph-BU-Cs Adsorbent

**Scheme 2:**
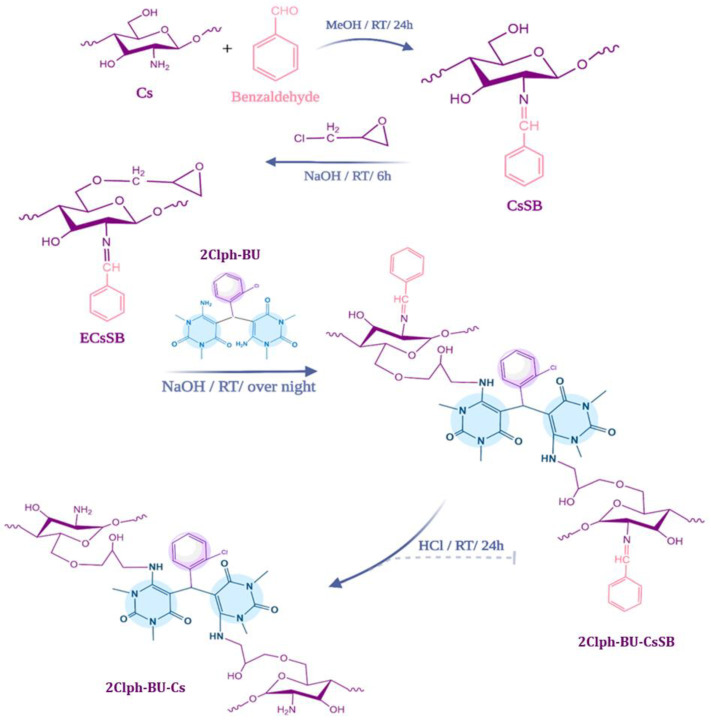
Synthesis of 2Clph-BU-Cs.

Step 1: Benzaldehyde (20 mL) was added slowly to the Cs suspension (5 g swollen in 60 mL of MeOH for 1 h) at RT and stirred for 24 h before being filtered. The formed yield (chitosan Schiff base, CsSB) was dried, after being washed several times with MeOH, for 8 h in an oven at 55 °C. This step was performed to protect the chitosan amino groups and confine the modification reaction on the primary -OH group at C6 in chitosan [23] (Scheme 2).

Step 2: Epichlorohydrin (10 mL) was slowly added to 4 g of CsSB that was stirred in 120 mL of aqueous NaOH solution (0.001 mol L^−1^) at RT for 20 min in order to swell and alkalize. The stirring was continued for an additional 6 h and the resulting epoxy chitosan Schiff base (ECsSB) was collected by filtration, rinsing many times with water then methanol and drying at 55 °C [16].

Step 3: Solution of 2Clph-BU (1.7 g, 1 mmol, in 25 mL of EtOH) was mixed with an ECsSB suspension (2.35 g, 2 mmol), that was swollen in aqueous NaOH solution (60 mL, 0.001 mol L^−1^), and the resultant mixture was stirred at RT overnight. The yield, 2Clph-BU-chitosan Schiff base (2Clph-BU-CsSB), was obtained through filtration, repeated washing with H_2_O and then MeOH, and finally drying at 55 °C.

Step 4: 2Clph-BU-CsSB (2 g) was stirred in ethanolic hydrochloric acid solution (60 mL, 0.24 mol L^−1^) at RT for 24 h. Then, 1 wt% aqueous Na_2_CO_3_ solution was used to neutralize the reaction mixture until pH 7 was reached. The resulted 2Clph-BU-chitosan (2Clph-BU-Cs) was dried to constant weight at 55 °C after being filtered and washed with water and ethanol (Scheme 2). 

### 2.3. Measurements

#### 2.3.1. Elemental Analysis

The C, H, N, S Analyzer (Perkin Elmer, Model 2410 series II, Waltham, MA, USA) was utilized to determine the elements of the Cs, CsSB, ECsSB, 2Clph-BU-CsSB, and 2Clph-BU-Cs.

#### 2.3.2. FTIR Spectroscopy

The FTIR spectroscopy measurements of the Cs, CsSB, ECsSB, 2Clph-BU-CsSB, and 2Clph-BU-Cs were recorded on Agilent Technologies FTIR Spectrometer (Cary 600 Series, Santa Clara, CA, USA) in the wavenumber range from 4000 to 400 cm^−1^.

#### 2.3.3. X-ray Diffractometry

An X-ray diffractometer (Joel JDX-8030, Tokyo, Japan) was utilized to investigate the morphology of Cs, CsSB, ECsSB, 2Clph-BU-CsSB, and 2Clph-BU-Cs at diffraction angles 2θ (degree) through a range from 5 to 90°, with a 5° min^−1^ speed.

#### 2.3.4. Scanning Electron Microscopy

A scanning electron microscope (Joel JSEM-6010PLUS/LV, Tokyo, Japan) was employed to image the surface topography of Cs, CsSB, ECsSB, 2Clph-BU-CsSB, and 2Clph-BU-Cs, after they have been coated with a gold, at a magnification of 5000 x and at a 15 kV accelerating voltage.

#### 2.3.5. Swelling Capability Study

The completely dried samples (10 mg) were immersed in buffer solutions of pH 4, 7, and 9 (100 mL) at 298, 308, and 328 K with shaking for varying times. Each sample was taken out of the immersion media and its weight was recorded after removing the excess solution droplets from its surface using filter sheet. According to Equation (1), the swell ability (%) was calculated [22].

(1) $$\%\mathrm{Swell}\;\mathrm{ability}=\frac{\left(\mathrm{Ws}\;-W_{0}\right)}{\;W_{0}}\times 100$$

where Ws and W_0_ are the swollen and the dry weight of the sample, respectively. Swelling capability measurements were performed many times and an average of three comparable results were taken.

#### 2.3.6. pH of Zero-Point Charge—pHzpc Study

The pHzpc was determined by soaking 0.1 g of 2Clph-BU-Cs for 24 h in 10 mL of (0.1 M NaCl) solution after adjusting its pH to a range from 3 to 12. The pH was adjusted using solutions of 0.1 N HCl and 0.1 N NaOH. Then, the pH of the solution was measured by a pH meter. The pHzpc value was obtained by plotting the graph of initial pH against ΔpH [32,33]. The pH measurements were performed many times and an average of three comparable results were taken.

### 2.4. Adsorption Behaviors

#### 2.4.1. Adsorption Capacity 

Ten milligrams of adsorbent (2Clph-BU-Cs) was added to 10 mL of a CR dye solution and agitated at 180 rpm in a water bath until equilibrium was achieved. The concentration of the dye solution was measured after removing the adsorbent using an ultraviolet–visible spectrophotometer (Shimadzu UV/Vis 1601 spectrophotometer, Japan, detection wavelength = 497 nm). 

Using Equations (2) and (3), the adsorption capacity (q, mg g^−1^) and the % removal efficiency (% RE) of the dye can be calculated, respectively [11].

(2) $$Q=\frac{\left(C_{1}-C_{2}\right)\;V}{m}$$

(3) $$\%\mathrm{RE}=\frac{\left(C_{1\;}-C_{2}\right)}{C_{1}}\times 100$$

where C_1_ and C_2_ (mg L^−1^) are the dye solution concentrations before and after adsorption, respectively, m is the adsorbent mass (g), and V (mL) is the volume of dye solution. 

The effect of contact time of the adsorbent, temperature (298–333 K), pH (4–9) and the initial dye solution concentration (30–120 mg L^−1^) were studied. All adsorption capacity experiments were performed many times and an average of three comparable results were taken.

#### 2.4.2. Adsorption Thermodynamics

To study the adsorption process, various thermodynamic parameters were investigated such as Gibbs free energy change $\Delta G^{0}\;$(kJ mol^−1^), entropy change ΔS° (J mol^−1^K^−1^), and enthalpy ΔH° (kJ mol^−1^) using Equations (4)–(6).

(4) $$\Delta G^{0}=-{\mathrm{RTln}\;K}_{C}$$

(5) $${\;\mathrm{lnK}}_{c}=\frac{\Delta S{}^{\circ}}{R}-\frac{\Delta H{}^{\circ}}{\mathrm{RT}}\;$$

(6) $${\;K}_{c}=\frac{q_{e}}{C_{e}}$$

where R (8.314 J mol^−1^K^−1^) is the constant of universal gas, T (K) is the absolute temperature, and K_c_ (L g^−1^) is the constant of standard thermodynamic equilibrium. By plotting the slope and intercept ln K_c_ vs. 1/T, the values of ΔS° and ΔH° can be calculated.

#### 2.4.3. Adsorption Kinetics 

The dye adsorption process’s kinetic properties were also thoroughly investigated. The dye solution concentration was measured at various times, and q_t_ the adsorption capacity at each time interval was determined using Equation (7).

(7) $$q_{t}=\frac{\left(C_{0\;}-C_{t}\right)\;V}{m}$$

where the dye concentrations at time t and the initial dye concentration are represented by C_t_ and $C_{0\;}$(mg L^−1^), respectively, V (mL) is the dye solution volume, and m (g) is the adsorbent mass. The pseudo-first-order model [34], pseudo-second-order model [35], Elovich model [10] and the Weber–Morris model of intraparticle diffusion [36] were utilized to analyze the adsorption capacity at various time intervals using Equations (8), (9), (10) and (11), respectively.

(8) $$\log\left(q_{e}-q_{t}\right)=\log q_{e}-\frac{k_{1}}{2.303}t$$

(9) $$\frac{t}{q_{t}}=\frac{1}{k_{2}q_{e}^{2}}+\frac{t}{q_{e}}$$

(10) $$q_{t}=\frac{1}{\beta }\ln\left(\alpha \beta \right)+\frac{1}{\beta }\ln t$$

(11) $$q_{t}=k_{\mathrm{int}}t^{\frac{1}{2}}+C$$

where the absorption capacities at a certain time t (min) and at equilibrium state are q_t_ and q_e_ (mg g^−1^), respectively, k_1_ (min^−1^) and k_2_ (g mg^−1^min^−1^) are the rate constants associated with the pseudo-first-order and the pseudo-second-order models, respectively, α (mg g^−1^ min^−1^) is the constant of initial adsorption rate, and β (g mg^−1^) is the constant of desorption related to the chemisorption activation energy and the surface coverage. The values of q_t_ and ln t can be found on a straight line by plotting them against each other, where values of α and β can be obtained. C (mg g^−1^) and k_int_ (mg g^−1^ min^−1/2^) are the intercept indicating the border layer’s thickness and the constant of rate associated with the intraparticle diffusion model, respectively. The slope of the q_t_ versus t^1/2^ plot can be used to calculate the value of k_int_.

#### 2.4.4. Adsorption Mechanism

The interaction between the adsorbent and dye molecules at equilibrium was described using the Langmuir [37], Freundlich [38], Temkin [4], and Dubinin–Radushkevich (D–R) [39], isotherm models using the Equations (12), (13), (14) and (15), respectively.

(12) $$\frac{C_{e}}{q_{e}}=\frac{1}{\left(q_{m}.K_{L}\right)}+\frac{C_{e}}{\left(q_{m}\right)}\;$$

(13) $${\;\mathrm{lnq}}_{e}=\ln K_{F}+\frac{1}{n}\ln C_{e}\;$$

(14) $$q_{e}={\mathrm{Bln}\;K}_{T}+B\ln C_{e}$$

(15) $${\mathrm{lnq}}_{e}=\ln q_{m}-K_{\mathrm{DR}}\varepsilon^{2}$$

where q_m_ and q_e_ (mg g^−1^) are the maximum adsorption capacity and adsorption capacity at equilibrium, respectively. The equilibrium dye solution concentration is C_e_ (mg L^−1^). The Langmuir constant K_L_ (L mg^−1^) relates to both the free energy and adsorption affinity. The Freundlich constants K_f_ (L mg^−1^) and n pertain to adsorption capacity and intensity, respectively. K_T_ is the binding constant of the Temkin isotherm, and B is the Temkin constant that is controlled by the adjusted uptake temperature (J mol^−1^). The constants B and K_T_ can be calculated from the slope and the intercept of a plot of q_e_ vs. ln C_e_. In the D-R model, K_DR_ (kJ^2^ mol^−2^) is related to the average adsorption free energy in the process of adsorption. ε value is the potential energy for Polanyi. Equation (16) defines the Langmuir separation factor (essential isotherm character and dimensionless; R_L_).

(16) $$R_{L}=\frac{1}{\left(1+K_{L}C_{o}\right)}$$

The R_L_ demonstrates the linear nature of isotherm (R_L_ = 1), irreversible (R_L_ = 0), favorable (0 < R_L_ < 1), and unfavorable (R_L_ > 1).

The value ε was calculated using Equation (17).

(17) $$\varepsilon =\mathrm{RTln}\;\left(1+\frac{1}{C_{e}}\right)$$

Equation (18) can be used to calculate K_DR_. The K_DR_ provides information about E (kJ mol^−1^), the mean free energy of adsorption per molecule of the adsorbate.

(18) $$E=\frac{1}{\sqrt{2K_{\mathrm{DR}}}}$$

The isotherm’s nature is indicated by the value of E (physisorption, E < 8 kJ mol^−1^) or (chemisorption, E = 8–16 kJ mol^−1^). 

#### 2.4.5. Desorption Study 

The adsorbent was washed with distilled water to remove any dye molecules that had not yet been adsorbed on its surface. Then, 0.01 g of the adsorbent was submerged in 10 mL of the desorption medium (methanol, ethanol, 0.1 N aqueous sodium hydroxide solution, or acetone) at 298 K for 24 h. Equation (19) can be used to calculate the amount of the desorbed dye.

% Dye desorption = q_d_/q_a_ × 100 (19)

where the quantity of desorbed dye from the adsorbent is q_d_ (mg g^−1^) and the quantity of dye absorbed by the adsorbent is q_a_ (mg g^−1^) [10]. 

## 3. Results and Discussion

### 3.1. Synthesis of 2Clph-BU-Cs Adsorbent

At the final stage of a reaction consisting of four consecutive steps, 2Clph-BU-Cs adsorbent (Scheme 2) was obtained. In the first step, Cs amine groups were protected by condensing them with the benzaldehyde C=O groups, producing CsSB. Hence, in the second step, the reaction with epichlorohydrin was confined on the primary hydroxyl groups at C6, creating ECsSB. Afterwards, epoxy rings of ECsSB, in the third step, were facilely opened using a nitrogen-rich 2Clph-BU via its lone pair of electrons, yielding 2Clph-BU-CsSB. At the end, in the fourth step, the amino groups of Cs were recovered by removal of the protection in an acidic medium, producing 2Clph-BU-Cs. Thus, the combination of a nitrogen-rich 2Clph-BU-Cs, as linkages between the Cs chains, hydroxyl groups, and the regained amino groups at Cs, will greatly enhance the cationic sites that possess high capacity to adsorb the acidic pollutants like CR dye.

### 3.2. Characterization of 2ClphBU-Cs Adsorbent

#### 3.2.1. Elemental Analysis

The elemental analysis values of all modified Cs derivatives are recorded in Table 1. In regard to %C and %N, it could be noted that the %C value of CsSB (62.80) increased at the expense of its %N value (5.70) compared to those of the virgin Cs (%C, 44.90 and %N, 8.61). This confirms the successful amalgamation of the carbon-rich benzaldehyde moieties into the repeating units of Cs. In contrast, the %N value of the 2Clph-BU-CsSB was 10.79, which was higher than that obtained for both CsSB (5.70) and ECsSB (4.48). This is due to nitrogen-rich 2Clph-BU moiety that was incorporated into the repeating units of ECsSB. This was additionally proved by the presence of chlorine element (3.42%) in the elemental analysis of 2Clph-BU-CsSB. Moreover, a decrease in the %C value accompanied by a further increase in the %N value of the 2Clph-BU-Cs (C, 51.14 and N, 13.00), in comparison to 2Clph-BU-CsSB (C, 58.62 and N, 10.79) was observed. This emphasizes the removing of the benzaldehyde moieties from Cs part and retrieving its 1^ry^ amino groups.

#### 3.2.2. FTIR Spectroscopy

**Figure 2:**
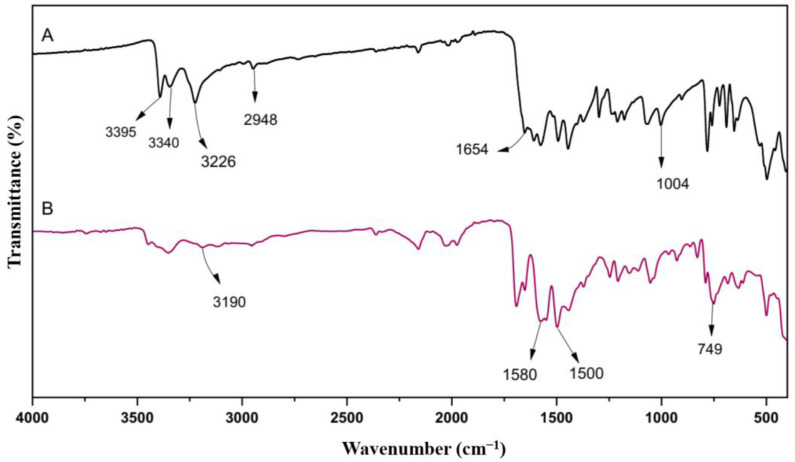
FTIR spectra of: (A) 6-amino-1,3-dimethyl uracil, and (B) 2Clph-BU.

The FTIR spectrum of 6-amino-1,3-dimethyl uracil (Figure 2) showed two absorption peaks corresponding to the -NH_2_ group at 3395 and 3340 cm^−1^. The peak at 3226 is due to N-H, and the peak at 2948 due to C-H stretching. The stretching vibration related to the C=O group and C-H of the pyrimidine moiety appeared at 1654 and 1004 cm^−1^, respectively [40]. 

Meanwhile, the peak of the C-H of the pyrimidine nucleus at 1004 cm^−1^ completely disappeared in the FTIR spectrum of 2Clph-BU (Figure 2), confirming its condensation with the carbonyl of the aldehyde group of the 2-chlorobenzaldehyde (Scheme 1). Additionally, there was the appearance of some new peaks at 3190 cm^−1^ (C-H aromatic) [4], at 1500 and 1580 cm^−1^ (C=C aromatic), and at 749 cm^−1^ (C-Cl). This indicates that 2Clph-BU had successfully been synthesized [41]. 

**Figure 3:**
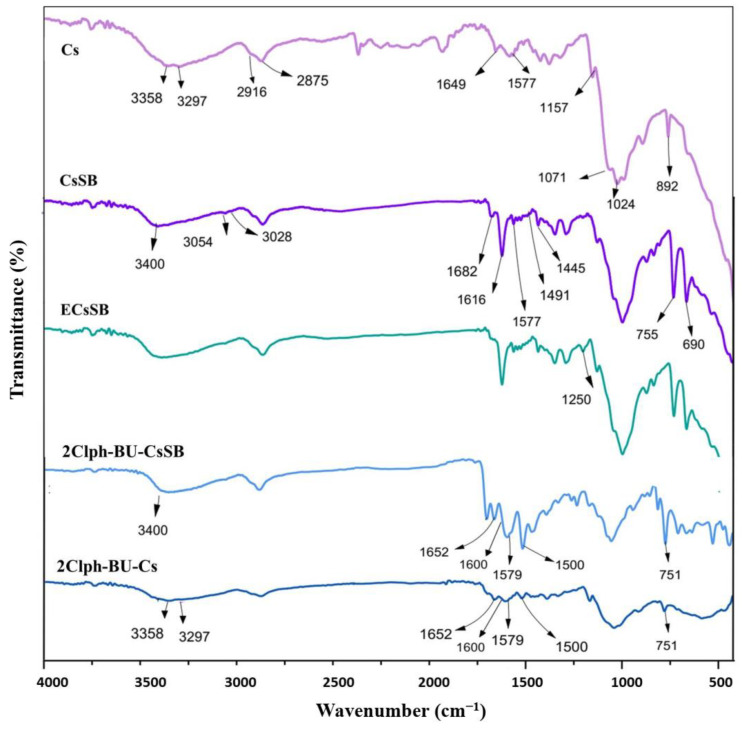
FTIR spectra of 2Clph-BU-CsSB and 2Clph-BU-Cs adsorbent.

On the other hand, Figure 3 shows the FTIR spectra of Cs before and after modification. Between 3700 and 3000 cm^−1^, a very broad absorption peak was observed, corresponding to the peak of hydroxyl groups overlapping with that of -NH_2_ groups and their hydrogen bonds. In this wavenumber range, there is a doublet peak at 3358 and 3297 cm^−1^ related to the -NH_2_ groups. The symmetric stretching vibration peaks of the -CH and -CH_2_ groups in the moieties of pyranose appeared at 2916 and 2875 cm^−1^, respectively. Further, two weak peaks appeared at 1649 and 1577 cm^−1^ attributable to amide I and amide II, respectively, confirming the high Cs deacetylation degree. The four absorption peaks that appeared at 1157, 1071, 1024, and 892 cm^−1^ in the spectrum of FTIR of virgin Cs confirmed its saccharide moieties [21].

In the CsSB spectrum, the doublet peak corresponding to the NH_2_ groups of chitosan disappeared and was replaced by a single one at 3400 cm^−1^, which can be attributed to -OH groups. In addition, some new peaks were observed; at 3054 and 3028 cm^−1^ (C-H, aromatic), at 1682 cm^−1^ (C=N groups), at 1616, 1577, 1491, and 1445 cm^−1^ (C=C, aromatic), and at 755 and 690 cm^−1^ (mono-substituted benzene ring) [4], indicating that all the -NH_2_ groups were consumed during protection with benzaldehyde and confirming the successful formation of CsSB.

In addition to the aforementioned stated peaks for CsSB, the ECsSB spectrum revealed a new peak at 1250 cm^−1^ that is attributed to the moieties of epoxide [25].

The spectrum of 2Clph-BU-CsSB revealed the evanescence of the peak at 1250 cm^−1^ that relates to the epoxy nucleus. This was accompanied by the appearance of new absorption peaks; at 1652 cm^−1^ (C=O, pyrimidine ring), at 1500, 1579, and 1600 cm^−1^ (C=C, aromatic ring), and at 751 cm^−1^ (C-Cl group), affirming the completeness of the reaction of ECsSB with 2Clph-BU. 

The restoring of the doublet peak of the -NH_2_ groups of chitosan moieties at 3358 and 3297 cm^−1^, in addition to the demise of the of mono-substituted benzene rings absorption bands at 755 and 690 cm^−1^ confirm the elimination of benzaldehyde nuclei to obtain 2Clph-BU-CS adsorbent.

#### 3.2.3. Powder X-ray Diffractometry

**Figure 4:**
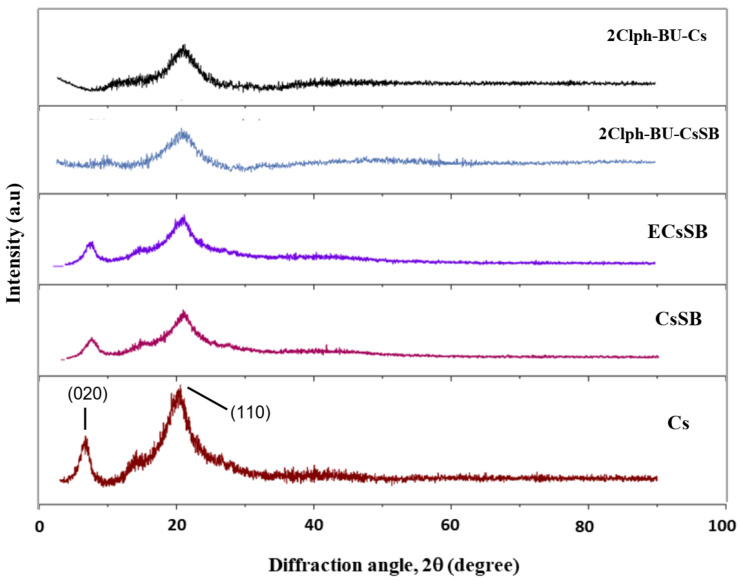
XRD patterns of 2Clph-BU-CsSB and 2Clph-BU-Cs adsorbent.

To inspect the changes in the internal structure of chitosan before and after its modification, X-ray diffractometry was utilized. The X-ray diffraction patterns of the virgin Cs and its modified derivatives are illustrated in Figure 4. In Cs, a distinguishable two peaks close to 2θ = 10° and 20° were observed related to its amine I (−NH−CO−CH_3_) and amine II (−NH_2_), and corresponding to the 020 and 110 planes, respectively, they are consistent to those previously reported [42]. This can be attributed to the creation of many hydrogen bonds throughout its chains as a result of the abundance of its hydroxyl and amino groups. In comparison to Cs, the CsSB, ECsSB are less crystalline. This was illustrated not only by lowering the intensity of both these peaks but also by increasing their broadening. Moreover, the complete disappearance of the peak at 2θ = 10° together with a further broadening and reduction in the intensity of the peak at 2θ = 20° were observed in both patterns of 2Clph-BU-CsSB and 2Clph-BU-Cs, suggesting their much lower crystallinity. The incorporation of the modifier linkages into the repeating units of Cs greatly decreased the possibility of formation of the hydrogen bonds between its chains. This is ascribed not only to the exhausting of its polar groups (-NH_2_ and/or -OH) during the modification process but also the separation of the Cs chains away from each other. This boosts the amorphous part and diminishes the crystalline zone. 

#### 3.2.4. SEM Analysis

**Figure 5:**
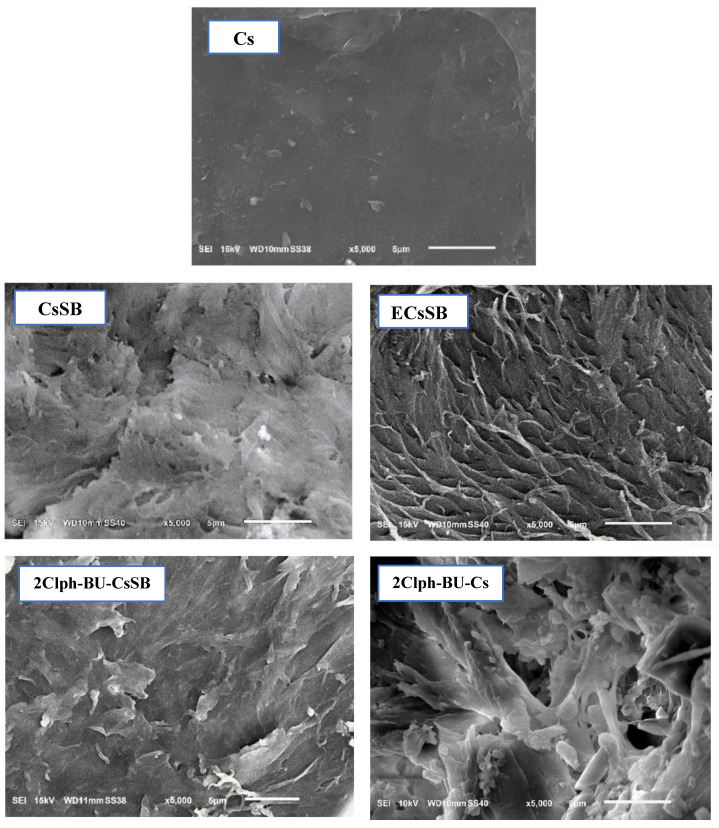
SEM images of 2Clph-BU-CsSB and 2Clph-BU-Cs adsorbent.

The SEM images of the topographical features of the surfaces of Cs, CsSB, ECsSB, 2Clph-BU-CsSB, and 2Clph-BU-Cs are shown in Figure 5. It was observed that Cs surface was very smooth, while its modified derivatives had much more rough surfaces that were composed of lumps of various sizes due to the size differences of the inserted modifier moieties. It can be also noted that the distribution of these lumps in each derivative was homogenous, meaning that the modification of Cs in each step was successfully accomplished.

#### 3.2.5. pH of Zero-Point Charge (pHzpc) 

**Figure 6:**
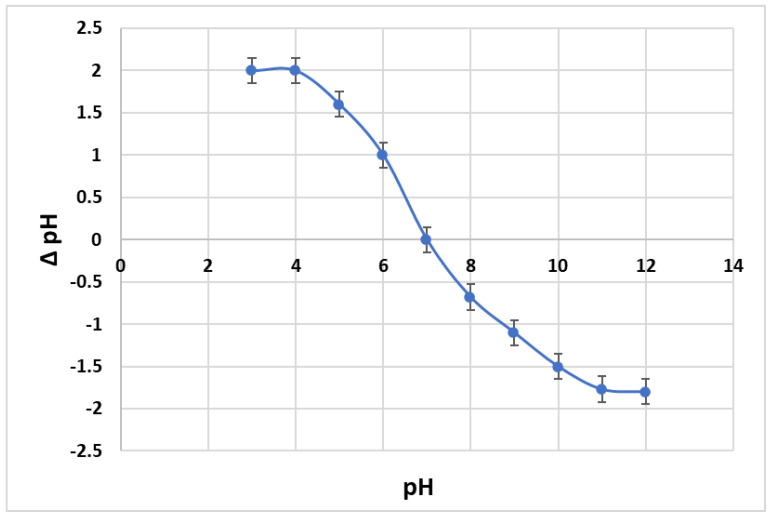
pH_zpc_. plot for 2Clph-BU-Cs.

The pH of zero-point charge is defined as the pH at which the surface net charge becomes equal to zero under certain conditions of temperature and aqueous solution composition. This does not imply that the surface has no charge at pHpzc, but rather that there are equal amounts of positive and negative charges. The magnitude of the surface charge depends on the abundance and types of functional groups, and on the pH of the solution. The pHzpc plays an important role in surface characterization as it dictates how easily an adsorbent can bind potentially harmful ions. This is due to the fact that the adsorbent surface bears a net negative charge at pH > pHzpc, making the adsorption of cationic species more favorable. Conversely, for pH < pHzpc value, the adsorbent surface bears a net positive charge $-{\mathrm{NH}}_{2}^{+}$ capable of repelling cations. The pHzpc value for 2Clph-BU-Cs obtained using the salt addition method was found to be 7 (Figure 6). The surface of the 2Clph-BU-Cs becomes more cationic with a decrease in pH value. This is attributed to the protonation of (-NH_3_^+^, -OH_2_^+^, -NH_2_^+^, C=OH^+^, and -HN^+^-Me) groups [32,33].

#### 3.2.6. Swelling Behavior of 2ClphBU-Cs 

**Figure 7:**
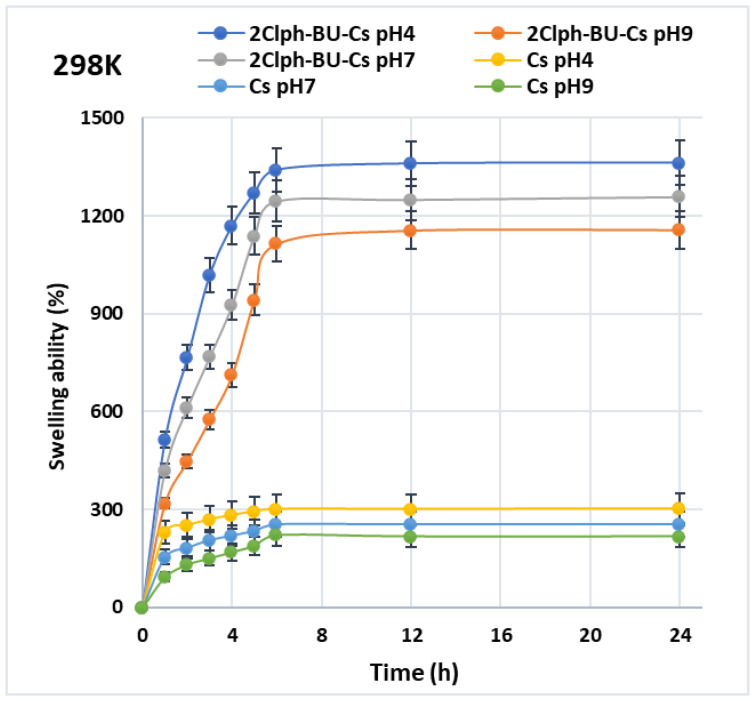
Swelling ability of Cs and 2Clph-BU-Cs hydrogel in pH 4, 7, and 9 at T = 298 K.

**Figure 8:**
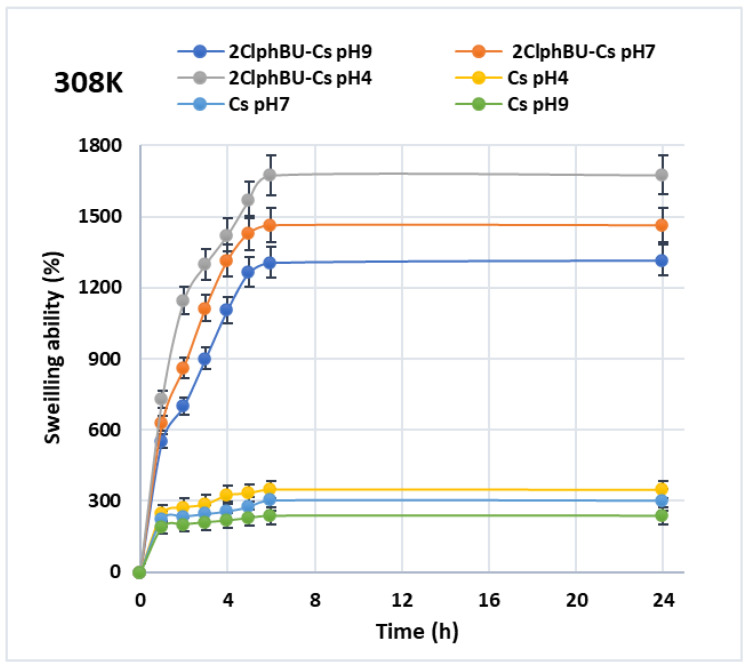
Swelling ability of Cs and 2Clph-BU-Cs hydrogel in pH 4, 7, and 9 at T = 308 K.

**Figure 9:**
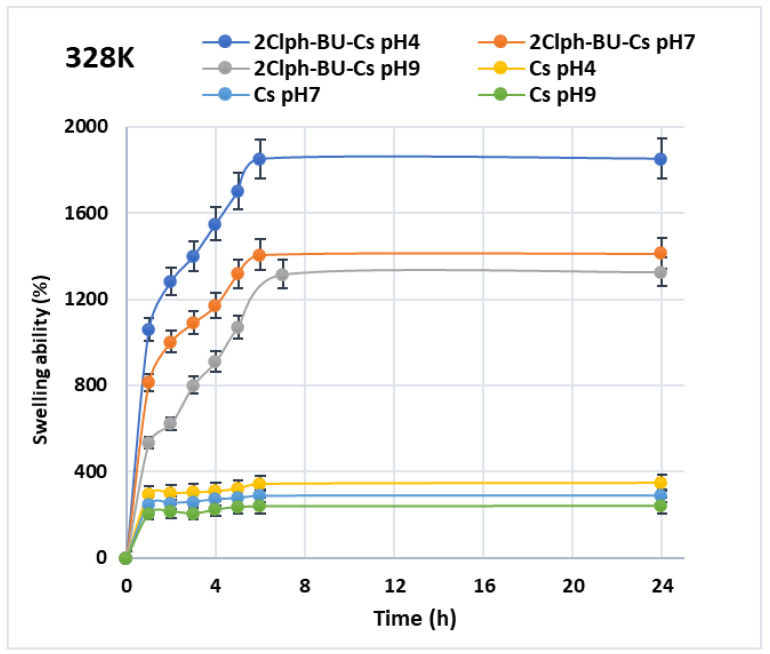
Swelling ability of Cs and 2Clph-BU-Cs hydrogel in pH 4, 7, and 9 at T = 328 K.

Figure 7, Figure 8 and Figure 9 show the swelling ability of Cs and 2Clph-BU-Cs adsorbent in pH 4, 7, and 9 at 298, 308, and 328 K until equilibrium is reached. The swelling capability exhibited by 2Clph-BU-Cs was higher than that obtained for the parent Cs at all the examined temperatures and pH values.

Chitosan is known as a pH-sensitive polymer, owing to its possession of numerous amino and hydroxyl groups which are easily protonated in acidic media. The repulsion forces between the resulting similarly charged mobile ions causes an osmosis imbalance that facilitates the diffusion of the liquid inside Cs matrix, increasing its swell ability. An increase in pH causes a decrease in the Cs swelling capacity owing to deprotonation of the amino and hydroxyl groups, dropping the repulsion between the chains and leading to its shrinkage [22]. Thus, Cs swells at pH 4 more than at pH 7 and 9. 

Furthermore, 2Clph-BU-Cs also showed a pH-sensitive character due to its ownership of nitrogen-rich bis-uracil moieties in addition to the regained amino groups and the voids generated from incorporation of 2Clph-BU crosslinker between the Cs chains. Thus, 2Clph-BU-Cs acts as a cationic biopolymeric hydrogel that significantly swells in acidic media. Also, the swelling ability of 2Clph-BU-Cs at pH 4 was higher than pH 7 and 9.

Further, both Cs and 2Clph-BU-Cs exhibited temperature-sensitive character; their swelling capability was greater with increased temperatures at all the pH levels used (Figure 7, Figure 8 and Figure 9). This is due to relaxation of the polymeric chains with rising temperatures increasing the electrostatic interaction, enhancing osmotic pressure inside the hydrogels, and permitting much water penetration into the hydrogel [43].

#### 3.2.7. Factors Affecting the Adsorption Process

To investigate the adsorption behavior of 2Clph-BU-Cs adsorbent for CR dye in more depth, the impacts of temperature, contact time, pH, and the initial dye concentration on adsorption capacity were studied.

**Figure 10:**
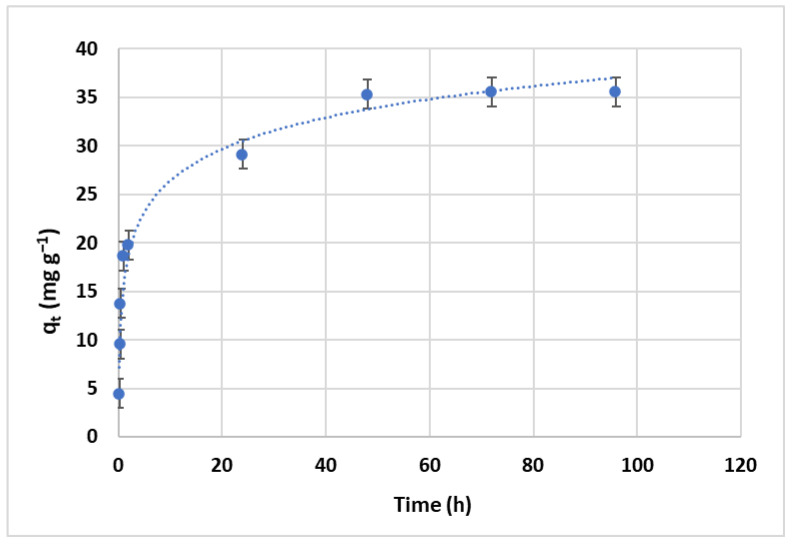
Effect of time on the adsorption capacity (m = 0.01 g, C_0_ = 90 mg L^−1^, V = 10 mL, pH = 4 and T = 298 K).

The contact time’s effect on adsorption capacity (Equation (7), m =0.01 g, C_0_ = 90 mg L^−1^, V = 10 mL, pH = 4 and T = 298 K) is depicted in Figure 10. The adsorption rate was rapid at first and then gradually tapered down until the adsorption equilibrium condition was reached. This is due to the fact that there are many active sites for adsorption on the adsorbent surface during the adsorption’s initial stages when the concentration of dye in the solution is high. Hence, in this stage, the adsorbent surface was mostly where adsorption occurred. The dye molecules had to diffuse into the adsorbent once the surface adsorption sites were filled; however, because of the mass transfer resistance they met throughout the diffusion process, the rate of adsorption gradually decreased.

**Figure 11:**
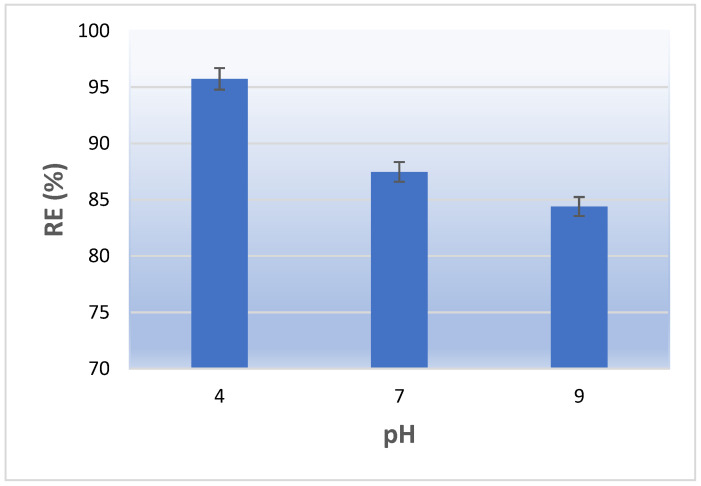
Effect of pH on the dye removal efficiency (m = 0.01 g, C_0_ = 50 mg L^−1^, V = 10 mL, T = 298 K, and time = 48 h).

The results of the effect of pH in the range of 4–9 of the dye solution on the absorption behavior (Equation (8), m = 0.01 g, C_0_ = 50 mg L^−1^, V =10 mL, T = 298 K, and time = 48 h) are shown in Figure 11. It is obvious that pH played an important role in the adsorption process, since the dye removal efficiency (% RE) in the acidic medium (pH = 4) was larger than that in both neutral and basic media (pH = 7 and 9). On one hand, in the acidic medium, the electrostatic interaction between the various protonated polar sites on the adsorbent (-NH_3_^+^, -OH_2_^+^, -NH_2_^+^, C=OH^+^, and -HN^+^-Me) and the anionic dye molecules increases. Furthermore, it enables dye molecules to interact in the relaxed network and diffuse into the adsorbent. This corresponds to the value of pHzpc which indicates that the surface of the adsorbent carries a positive charge at low pH values.

**Figure 12:**
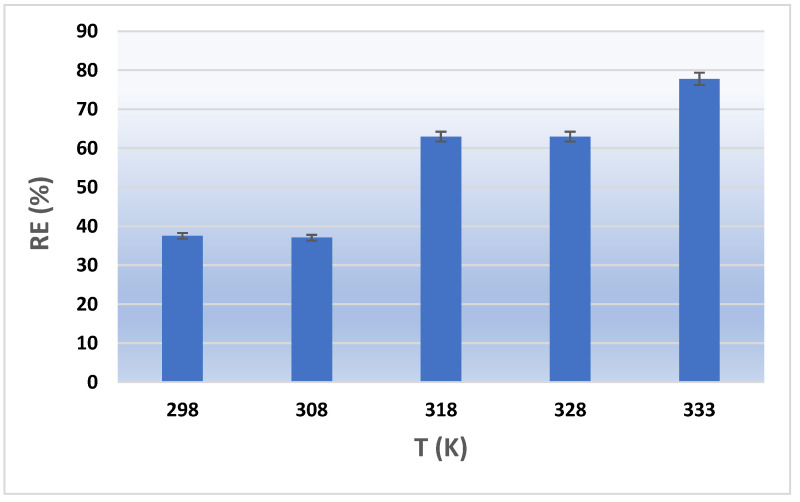
Effect of temperature on the dye removal efficiency (m = 0.01 g, C_0_ = 90 mg L^−1^, V = 10 mL, pH = 4, and time = 48 h).

Figure 12 shows the effect of temperature (298–333 K) on the dye removal efficiency (Equation (8), m = 0.01 g, C_0_ = 90 mg L^−1^, V = 10 mL, pH = 4, and time = 48 h). It was found that the removal efficiency increased gradually with an increase in temperature. Thus, an increased temperature is beneficial for the polymeric chains’ extension in the adsorbent with the formation of several gaps between them, which is beneficial to efficacy of the dye adsorption. Additionally, as the temperature rises, both the dye’s solubility and the dye molecules’ Brownian motion quicken, which enhance the dye molecules’ migration and diffusion into the adsorbent. Despite the fact that the adsorption process is an endothermic reaction, the system’s entropy actually rises during the process. Therefore, increasing temperature is advantageous for the adsorption process according to the Gibbs–Helmholtz Equation, as seen in Table 2.

**Figure 13:**
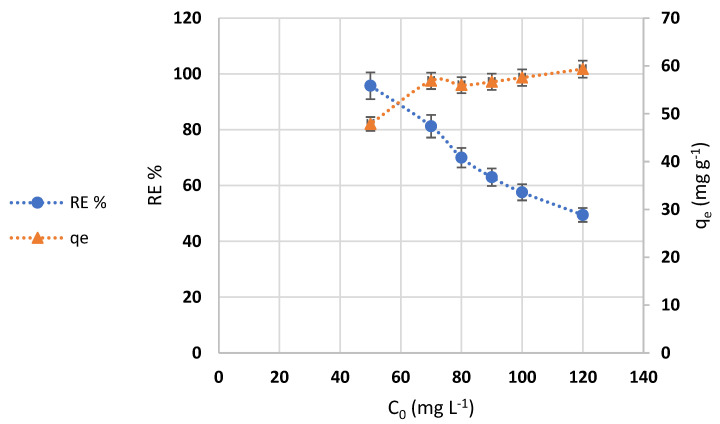
Initial concentration effect of the dye on the removal efficiency and the adsorption capacity (m = 0.01 g, V = 10 mL, pH = 4, T = 328 K, and time = 48 h).

To improve the absorption behavior of an absorbent, the dye’s initial concentration is another crucial factor. Figure 13 depicts how the dye concentration affected the capacity for adsorption and the effectiveness of removal. The adsorption capacity grew whereas the removal efficiency dropped with an increase in dye concentration. The % RE is more than 80% when the concentration of the CR dye is low (70 mg L^−1^). Therefore, the findings show that the studied adsorbent has outstanding CR dye removal performance in practical applications.

The hydrogel 2Clph-BU-Cs showed a higher adsorptive capacity compared with the Cs and its other prepared derivatives. The values of % RE of CR dye (dye solution (10 mL, 30 mg L^−1^), pH 4, temperature 298 K, and adsorbent dose 10 mg) for Cs, CsSB, ECsSB, and 2Clph-BU-CsSB were 39.70%, 25.44, 29.80, and 34.9 %, respectively, lower than that of 2Clph-BU-Cs (54%). This indicates the important role played by both the amino groups and 2Clph-BU moieties at 2Clph-BU-Cs in the adsorption process.

#### 3.2.8. Adsorption Thermodynamics 

**Figure 14:**
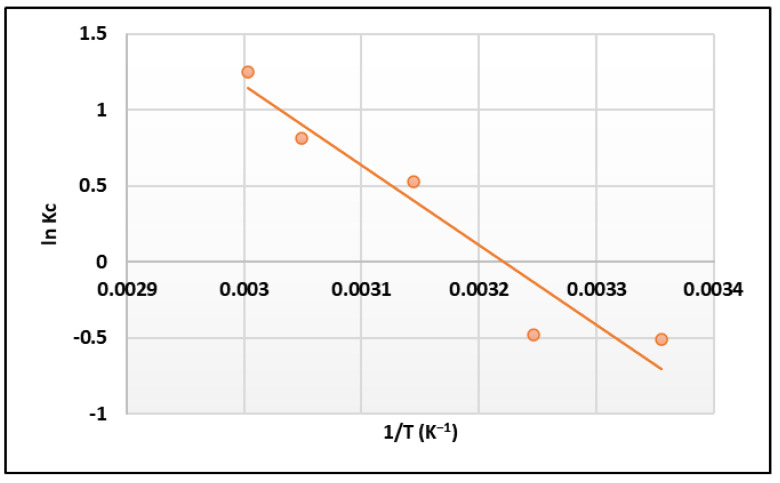
CR dye adsorption thermodynamic onto 2Clph-BU-Cs adsorbent.

Various thermodynamic characteristics, which are shown in Table 2 and Figure 14, were examined for further research of the adsorption process. According to the results obtained, the process was not spontaneous and unfavorable, as indicated by the positive values of G° at low temperatures. Nevertheless, at higher temperatures, it displayed negative values, indicating that the adsorption process was spontaneous and advantageous [4]. The positive values of ΔH° and ΔS° indicate that the adsorption process is endothermic with confusiondegree increasing process. 

#### 3.2.9. Adsorption Kinetics

**Figure 15:**
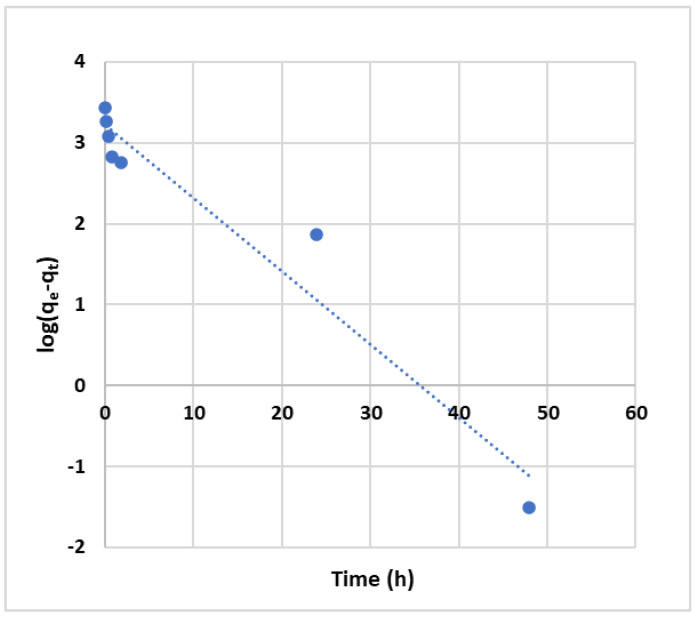
The model plot of pseudo-first-order for the CR dye adsorption onto 2Clph-BU-Cs adsorbent (m = 0.01 g, C_0_ = 100 mg L^−1^, V = 50 mL, pH = 4, and T = 298 K).

**Figure 16:**
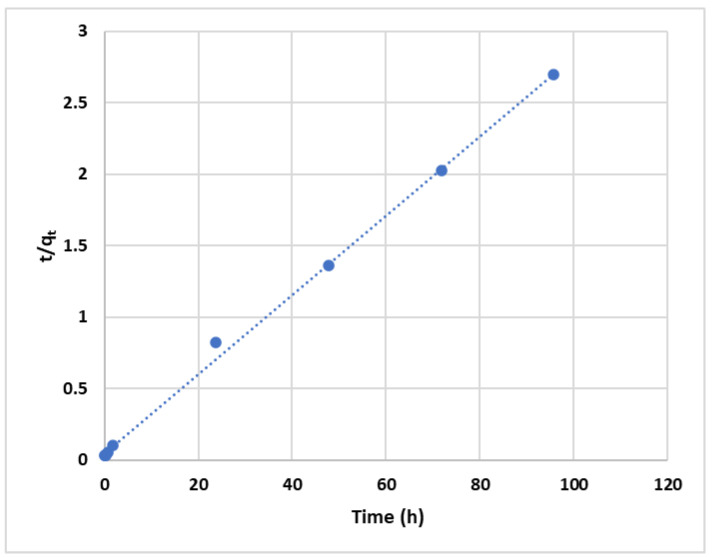
The model plot of pseudo-second-order for the adsorption CR dye onto 2Clph-BU-Cs adsorbent (m = 0.01 g, C_0_ = 100 mg L^−1^, V = 50 mL, pH = 4, and T = 298 K).

**Figure 17:**
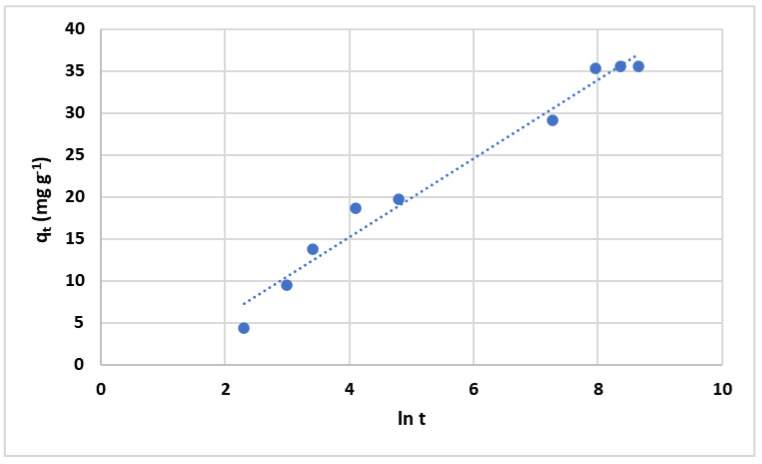
The model plot of Elovich for the adsorption of CR dye onto 2Clph-BU-Cs adsorbent (m = 0.01 g, C_0_ = 100 mg L^−1^, V = 50 mL, pH = 4, T = 298 K, and time = 48 h).

**Figure 18:**
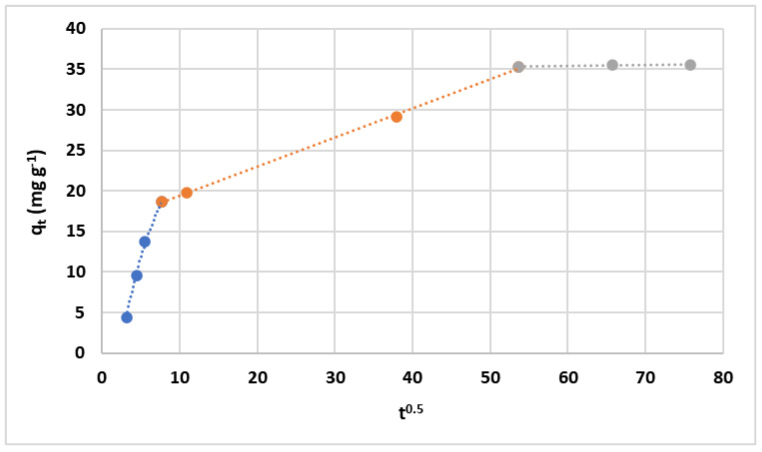
The 2Clph-BU-Cs adsorbent’s intraparticle diffusion model for the adsorption of CR dye (m = 0.01 g, C_0_ = 100 mg L^−1^, V = 50 mL, pH = 4, T = 298 K, and time = 48 h).

Figure 15, Figure 16, Figure 17 and Figure 18 illustrate the use of the pseudo-first-order, pseudo-second-order, Elovich, and Weber–Morris intraparticle diffusion models to investigate the adsorption behavior of CR dye onto 2Clph-BU-Cs adsorbent. The correlation coefficient (R^2^) of the pseudo-first-order kinetic model is smaller than that of the pseudo-second-order kinetic and Elovich models (Table 3). This leads to the conclusion that the absorption behavior is better described by a pseudo-second-order kinetic process. This suggests that electrostatic attraction and π-π interaction are involved in the chemisorption process in this adsorption system between the adsorbent and dye [44]. A linear relationship between q_t_ and t^1/2^ revealed that the adsorption process was restricted by intraparticle diffusion, according to the Weber–Morris model (Figure 18) [45]. Additionally, as shown in Figure 18, a three-stage adsorption pattern was seen, and the Weber–Morris model was unable to pass through the origin. These results indicate that a multi-step mechanism controls the adsorption process. [4]. The first stage (k_int_1), with a steeper slope in Figure 18, demonstrates that the rate-determining step is surface diffusion, which is the diffusion of the dye from the bulk solution to the boundary layer surrounding the adsorbent or to the external surface of the adsorbent through the boundary layer. The second stage (k_int_2) represents the stage of adsorption, where intraparticle or pore diffusion is another rate-determining step. All active sites on the adsorbent surface are saturated in the third stage (k_int_3) (equilibrium stage). These results indicate that the CR dye was present both inside and outside of the adsorbent’s structure [4].

#### 3.2.10. Adsorption Mechanism

**Figure 19:**
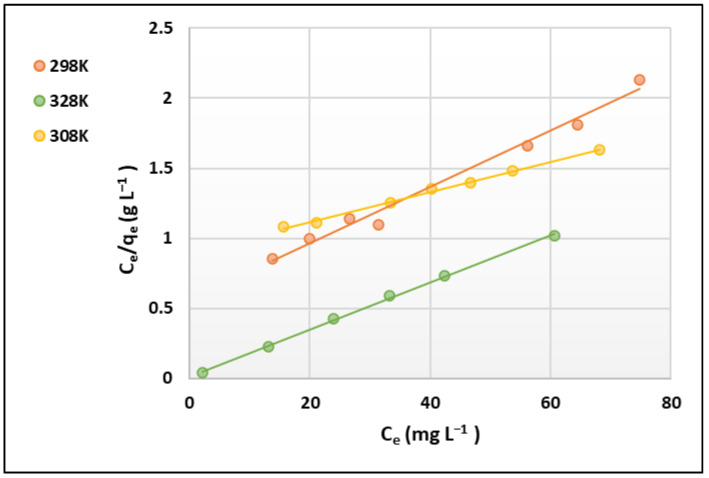
Langmuir isotherm plots for adsorption of CR dye onto 2Clph-BU-Cs adsorbent (m = 0.01 g, C_0_ = 30–120 mg L^−1^, V = 100 mL, pH = 4, and time = 48 h).

**Figure 20:**
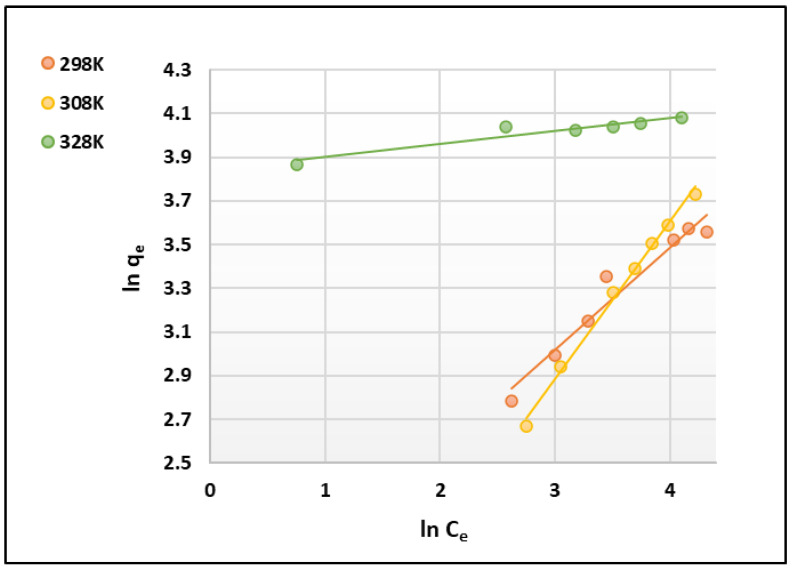
The plots of Freundlich isotherm for CR dye adsorption onto 2Clph-BU-Cs adsorbent (m = 0.01 g, C_0_ = 30–120 mg L^−1^, V = 100 mL, pH = 4, and time = 48 h).

**Figure 21:**
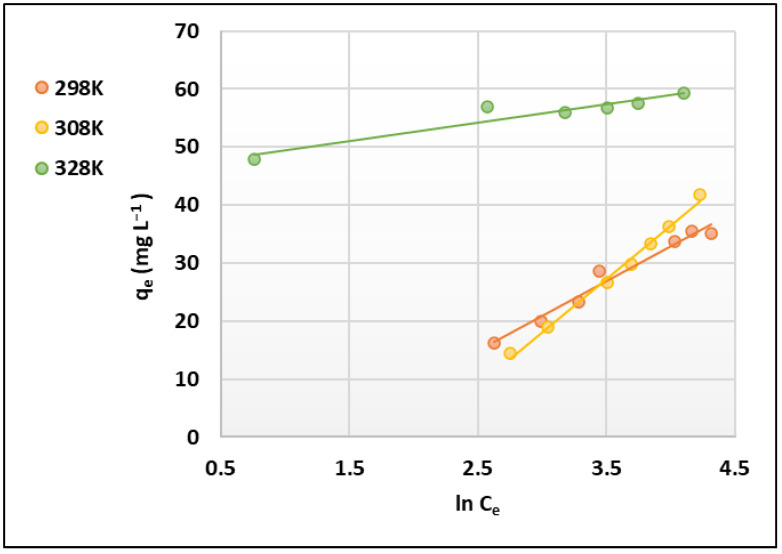
The plots of Temkin isotherm for CR dye adsorption onto 2Clph-BU-Cs adsorbent (m = 0.01 g, C_0_ = 30–120 mg L^−1^, V = 100 mL, pH = 4, and time = 48 h).

**Figure 22:**
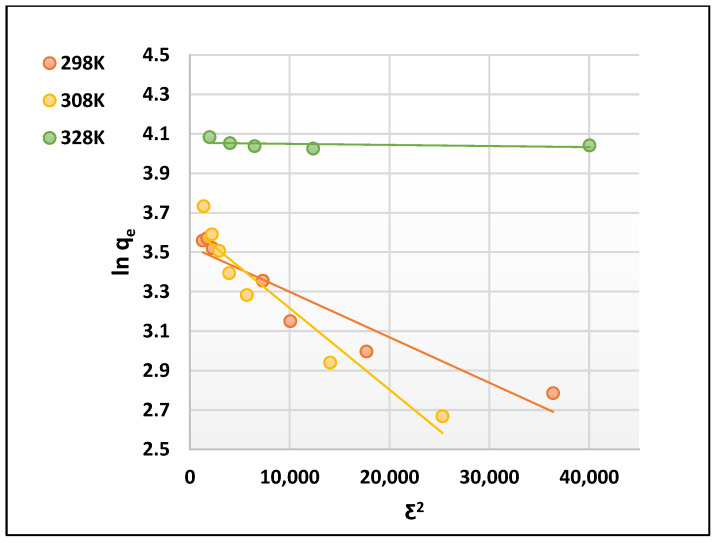
D-R isotherm plots for adsorption of CR dye onto 2Clph-BU-Cs adsorbent (m = 0.01 g, C_0_ = 30–120 mg L^−1^, V = 100 mL, pH = 4, and time = 48 h).

According to the Langmuir model, adsorption can occur on the surface of the adsorbent in equal amounts and the same binding site is used, where each adsorption molecule linked to the adsorbent surface has the identical adsorption energy [46]. The isotherm of the Freundlich model is obtained by supposing a heterogeneous surface with a nonuniform division of heat of adsorption over the surface via a multilayer adsorption procedure [47]. The Temkin model factored in the impact of the adsorbate molecules’ indirect interactions assuming that the adsorption heat of every molecule in the layer falls linearly as a result of interactions between adsorbent and adsorbate, The distribution of binding energies throughout the adsorption process is uniform [48]. The Polanyi theory is the basis of the D-R model. The adsorption system’s many adsorption mechanisms can be identified using this model [47]. The results of the investigation of the adsorption mechanism using the Langmuir, Freundlich, Temkin, and D-R models are shown in Figure 19, Figure 20, Figure 21 and Figure 22 and Table 4. It was discovered that the Freundlich model’s correlation coefficient (R^2^) was smaller than that of the Langmuir model, indicating that the Langmuir model was preferable to describe the adsorption process. Thus, the monolayer adsorption process dominates the absorption process. The separation constant (R_L_) can be determined using the Langmuir coefficient K_L_ to describe the adsorption reaction’s difficulty level. The R_L_ values are in the range of 0.41–0.77. Thus, the adsorption process can occur quite easily. Figure 22 depicts the plots of ln q_e_ vs. ε^2^ in the D-R adsorption model. The adsorption free energy (E) was also measured. In this condition, the E of adsorption are 0.159, 0.112, and 0.909 kJ mol^−1^, suggesting that physical interaction is dominant in the adsorption process [47].

**Figure 23:**
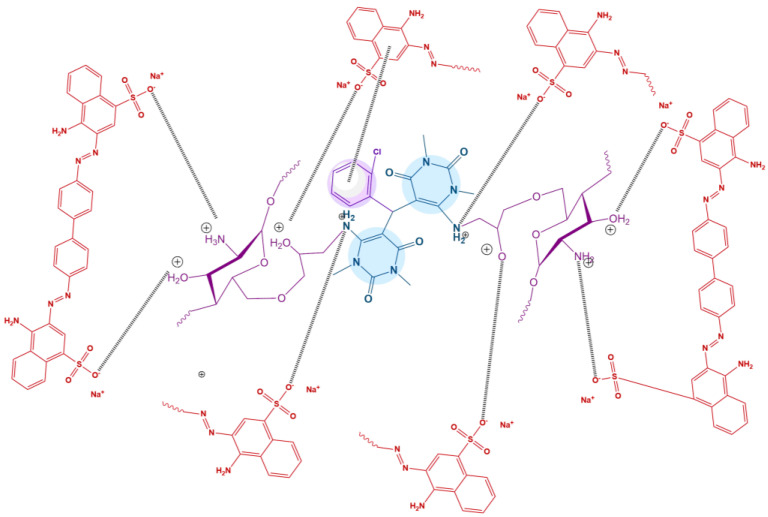
Schematic illustration of the interaction of CR dye with 2Clph-BU-Cs adsorbent.

Figure 23 provides a sketch of the adsorption mechanism. Thus, the excellent adsorption characteristics are a result of the dye and adsorbent electrostatic interaction, particularly for quick adsorption in the initial stage of adsorption. Moreover, the adsorption mechanism may be significantly influenced by the hydrogen bonding between both the functional groups on the dye molecules and those on the adsorbent [49]. 

#### 3.2.11. Comparison between 2Clph-BU-Cs and Other Adsorbents to Remove CR dye

It was interesting to compare the RE (%) for anionic CR dye of the 2Clph-BU-Cs adsorbent with the other previously reported adsorbents, as shown in Table 5, in order to analyze the adsorbent effectiveness. It is obvious that, compared to other adsorbents described in the literature, 2Clph-BU-Cs has the highest RE (%) for CR dye.

#### 3.2.12. Desorption and Regeneration Studies 

In a 0.1 N aqueous NaOH solution, CR dye was desorbed from the 2Clph-BU-Cs adsorbent, and the dye desorption was calculated using Equation (19). The dye desorption % reached 45% and 23% at the first and second cycles, respectively. While utilizing ethanol, methanol, and acetone as the desorption medium, no desorption was achieved. These results support the electrostatic mechanism through which CR dye is adsorbed to the 2Clph-BU-Cs adsorbent. 

## 4. Conclusions

Chitosan was chemically modified by incorporating 2Clph-BU linkages between its chains. The resultant 2Clph-BU-Cs adsorbent possesses -NH_2_ and -OH groups in addition to oxygen- and nitrogen- rich 2Clph-BU nuclei of the modifier, as confirmed by elemental analysis, FTIR, SEM, and XRD techniques. The removal efficiency of the anionic CR dye using 2Clph-BU-Cs hydrogel depended on temperature, pH, time, and initial dye concentration. The pseudo-second-order model accurately described the adsorption process, which suggests chemisorption behavior. The considerably strong correlation coefficient R^2^ of the Elovich model demonstrates that it correctly predicted the experimental findings for the adsorbent, indicating chemisorption process. Moreover, it was discovered by intraparticle diffusion that it was not the only process that was rate limiting. The adsorption isotherm can be well fitted to the Langmuir model indicating that the adsorbed dye molecules do not interact, and the adsorption process proceeds in a monolayer coverage manner. At 308 K, q_max_, which reached the maximum monolayer coverage capacity, was 93.46 mg g^−1^. The thermodynamic results suggest that the adsorption was endothermic in nature, spontaneous at higher temperatures, and non-spontaneous at low temperatures and also implies an increase in randomness at the interface between adsorbent and adsorbate solution.

## Figures and Tables

**Table 1:** Elemental analysis of the modified Cs derivatives.

Sample Code	Elemental Analysis				
	% C	% H	% N	% O	% Cl
Cs	44.90	6.86	8.61	39.63	-
CsSB	62.80	5.98	5.70	25.52	-
ECsSB	63.08	6.11	4.48	26.33	-
2Clph-BU-CsSB	58.62	5.65	10.79	21.52	3.42
2Clph-BU-Cs	51.14	5.83	13.00	25.80	4.23

**Table 2:** Adsorption thermodynamic parameters of CR dye onto 2Clph-BU-Cs adsorbent (m = 0.01 g, C_0_ = 90 mg L^−1^, V = 10 mL, pH = 4, and time = 48 h).

ΔH° (kJ mol^−1^)	ΔS° (J mol^−1^K^−1^)	ΔG° (kJ mol^−1^)					R^2^ (ln K_c_ against 1/T)
		298 K	308 K	318 K	328 K	333 K	
43.70	140.76	1.26	0.86	−1.45	−2.23	−3.47	0.923

**Table 3:** Constants of kinetic models and coefficients of correlation for the 2Clph-BU-Cs adsorbent’s adsorption of the CR dye (m = 0.01 g, C_0_ = 100 mg L^−1^, V = 50 mL, pH = 4, T = 298 K, and time = 48 h).

Kinetic Models	Parameters		
q_e_._exp_ (mg g^−1^)			35.56
pseudo- first-order	R^2^		0.942
	q_e_._cal_ (mg g^−1^)		0.15
	k_1_ (10^−4^)(min^−1^)		16.1
pseudo- second-order	R^2^		0.998
	q_e,cal_ (mg g^−1^)		36.10
	k_2_ (10^−4^)(g mg^−1^ min^−1^)		2.83
Elovich	R^2^		0.976
	β (g mg^−1^)		0.21
	α (mg g^−1^ min^−1^)		1.66
Intraparticle Diffusion	1st	K_int_	16.12
		R^2^	0.996
	2nd	K_int_	0.36
		R^2^	0.999
	3rd	K_int_	0.01
		R^2^	0.793

**Table 4:** Adsorption isotherm constants of CR dye by 2Clph-BU-Cs adsorbent (m = 0.01 g, C_0_ = 30–120 mg L^−1^, V = 100 mL, pH = 4, and time = 48 h).

Isotherm Mode	Parameters	298 K	308 K	l328 K
Langmuir	R^2^	0.985	0.996	0.999
	K_L_ (L mg^−1^)	0.01	0.01	1.11
	q_m_	49.75	93.46	59.52
	R_L_	0.48–0.77	0.44–0.74	0.41–0.63
Freundlich	R^2^	0.953	0.995	0.903
	K_f_	4.96	2.07	46.52
	1/n	0.47	0.71	0.05
Temkin	R^2^	0.970	0.992	0.905
	K_T_ (L mg^−1^)	0.28	0.13	2,268,261
	B (J mol^−1^)	12.05	18.32	3.16
D-R	R^2^	0.896	0.924	0.937
	q_m_ (mg g^−1^)	34.11	37.72	57.39
	$K_{\mathrm{DR}}$(mol^2^ J^−2^)	2 × 10^−5^	4 × 10^−5^	6 × 10^−7^
	E (kJ mol^−1^)	0.159	0.112	0.909

**Table 5:** Comparing 2Clph-BU-Cs with various adsorbents to remove CR dye.

Adsorbent	Removal Efficiency (%)	Temperature °C	Dye Concentration mg L^−1^	Adsorbent Dose (g)	pH	Ref.
Chitosan and Laponite based nanocomposite	78.06	30	500	1	6	[50]
GO/Chitosan	84.6	15	50	0.05	-	[51]
Lignin/Chitosan	86.5	35	200	0.01	3	[52]
Padina, gymnospora Zeolite	78.89	25	20	0.02	7	[53]
Water hyacinth roots	46.15	25	20	1	6	[54]
2Clph-BU-Cs	95.73	55	50	0.01	4	This study

## Data Availability

The data presented in this study are available on request from the corresponding authors.
